# A low-input Micro-C protocol for high-resolution 3D genome mapping

**DOI:** 10.1093/biomethods/bpag019

**Published:** 2026-04-15

**Authors:** Fengnian Shan, Chongren Pei, Sijian Xia, Fei Ling

**Affiliations:** School of Biology and Biological Engineering, South China University of Technology, Guangzhou 510006, China; Institute of Molecular Physiology, Shenzhen Bay Laboratory, Shenzhen 518132, China; Institute of Molecular Physiology, Shenzhen Bay Laboratory, Shenzhen 518132, China; Institute of Molecular Physiology, Shenzhen Bay Laboratory, Shenzhen 518132, China; School of Biology and Biological Engineering, South China University of Technology, Guangzhou 510006, China

**Keywords:** Micro-c, chromatin architecture, nucleosome resolution

## Abstract

Standard Micro-C protocols typically require millions of cells, limiting their application to rare cell populations. Here, we present an optimized low-input Micro-C workflow that requires only 100 000 cells. By downsampling both our low-input dataset and a control dataset from 5 million cells to 120 million raw read pairs, we demonstrate that all key architectural features—Compartments, Topologically associating domains (TADs), and Chromatin loops—are reliably detected from as few as 100 000 cells. The low-input protocol achieved a high cis interaction ratio (96.1%) and low PCR duplication rate (3.0%), indicating high library complexity and low background noise. Applying this method to investigate acute CTCF (CCCTC-binding factor) degradation, we observed the loss of loops and TAD boundaries in CTCF-degraded samples, consistent with previous reports. Our optimized protocol enables nucleosome-resolution 3D genome mapping for sample-limited studies.

## Introduction

The 3D organization of chromatin within the eukaryotic nucleus is fundamental to genome function. Far from being randomly arranged, genomic DNA is hierarchically folded into compact chromatin, establishing a highly ordered 3D architecture that plays critical roles in gene regulation, DNA replication, and genomic stability maintenance [1–8]. Elucidating the principles of this spatial organization has therefore become a central goal in modern molecular biology.

To this end, technological advances in chromosome conformation capture have enabled genome-wide interrogation of 3D chromatin architecture at progressively increasing resolutions. Hi-C, the most widely adopted approach, relies on restriction enzymes to digest cross-linked chromatin, generating fragments typically exceeding 1 kb kilobase [9]. This resolution limit restricts Hi-C’s ability to resolve fine-scale chromatin features. Micro-C was developed to overcome this limitation: it employs micrococcal nuclease (MNase) in place of restriction enzymes, cleaving linker DNA between nucleosomes to generate nucleosome-sized fragments (∼150–200 bp) [10]. This fundamental difference enables Micro-C to achieve nucleosome-resolution mapping, capturing not only long-range loops but also fine-scale interactions within compacted chromatin fibers that remain inaccessible to conventional Hi-C [11–18].

Following its initial development, subsequent methodological refinements have further enhanced Micro-C performance. The introduction of long crosslinkers such as DSG or EGS has been shown to reduce background noise and improve the capture efficiency of authentic long-range interactions [19]. Additionally, integration with ChIP-seq has enabled simultaneous profiling of chromatin conformation and protein–DNA binding from the same sample [20]. However, despite these advances, most current Micro-C protocols still require millions of cells as starting material [12, 20–25]. This high input requirement substantially restricts their application to precious clinical specimens or rare cell populations isolated by flow cytometry, where biological insights are most urgently needed.

To address this critical limitation, we report an optimized low-input Micro-C protocol that robustly generates high-quality data from as few as 100 000 cells. Through systematic MNase titration and simplification of the ligation step to a one-step reaction, we achieve efficient nucleosome fragmentation and library construction. Comprehensive benchmarking against conventional high-input Micro-C demonstrates faithful recapitulation of compartments, TADs, and loops. We further validate its biological utility through acute CTCF degradation experiments, recapitulating the loss of loops observed in previous reports [26]. This optimized workflow broadens the applicability of nucleosome-resolution 3D genome mapping to sample-limited research.

## Materials and methods

### Cell culture

G1E-ER4 cells and their derivative cell line, CTCF-AID-HaloTag, were cultured in IMDM medium. The medium was supplemented with 15% fetal bovine serum, 2% penicillin–streptomycin solution, 50 ng/ml mouse stem cell factor, 7.5 ng/ml Epogen, and 0.001% 1-Thioglycerol. All cell lines were routinely tested for mycoplasma contamination and maintained at 37 °C in a humidified atmosphere of 5% CO_2_.

### MNase digestion

For each low-input sample (100 000 cells), cells were resuspended in 500 μl of 1% formaldehyde and incubated at room temperature for 10 min. The reaction was quenched with glycine at a final concentration of 1 M. Cells were then pelleted by centrifugation at 4°C and gently washed once with PBS using low-retention microcentrifuge tubes to minimize cell loss. The cells were subsequently subjected to a second cross-linking step using 3 mM EGS crosslinker (MCE, HY-130458) at room temperature for 30 min, followed by quenching with 1 M glycine. After a final PBS wash under the same low-retention, 4°C centrifugation conditions, the cells were lysed on ice with cell lysis buffer (10 mM Tris, pH 8.0, 10 mM NaCl, 0.2% NP-40) for 20 min. Chromatin was then digested with micrococcal nuclease (MNase, NEB, M0247S). The optimal MNase concentration was empirically determined for each new enzyme batch to achieve a prominent mononucleosomal band (∼150 bp) with minimal high-molecular-weight smear; a representative digestion condition was a 1:500 dilution of the stock enzyme with a 30 min incubation.

### T4 ligase ligation

After chromatin fragmentation by MNase, a ligation master mix was prepared containing the following components: 6 μl of 10× T4 ligase buffer (Thermo, B69), 2.5 μl of 1 mM biotin-dATP (Active-Motif, 14139), 2.5 μl of 1 mM biotin-dCTP (MCE, HY-D1668), 2.5 μl of 1 mM dGTP, 2.5 μl of 1 mM dTTP, 3 μl of 100 mM ATP (Thermo, R1441), 1.33 μl of T4 polynucleotide kinase (10 U/μl, NEB, M0201L), 1.33 μl of Klenow fragment (5 U/μl, NEB, M0210L), 1.33 μl of T4 DNA ligase (30 U/μl, Thermo, EL0013), 0.6 μl of BSA (20 mg/ml, BBI, B600036-0001), and 0.3 μl of 500 mM EGTA. The mixture was brought to a final volume of 60 μl with nuclease-free water.

The cell pellet was resuspended in the master mix and incubated sequentially: first at 25 °C for 2 h, then at 37 °C for 2 h, and finally at 16 °C for at least 8 h to ensure efficient ligation. After proximity ligation, unligated DNA ends were digested with Exonuclease III (NEB, M0206L) at 37 °C to reduce background noise in subsequent sequencing.

### Reverse cross-linking and DNA extraction

The sample was normalized to a final volume of 100 μl with ddH_2_O. The reaction was supplemented with 12.5 μl of Proteinase K (20 mg/ml), 12.5 μl of 10% SDS, and 1 μl of RNase A (10 mg/ml), yielding final concentrations of 2.5 mg/ml, 1.25%, and 0.1 mg/ml, respectively. Following incubation at 37°C for 30 min, the samples were heated at 65°C for at least 3 h to reverse cross-links. DNA was then purified using 0.8 volumes of DNA purification beads according to the manufacturer’s instructions and eluted for library construction.

### Biotin pull-down

Fifteen microliter of Dynabeads Myone Streptavidin C1 beads (Thermo, 65002) were placed on a magnetic stand and washed three times with BW buffer (Binding and Washing buffer, 5 mM Tris–HCl pH 7.5, 0.5 mM EDTA, 1 M NaCl). The beads were then resuspended in 50 μl of 2× BW buffer, followed by the addition of 50 μl of purified DNA. The mixture was incubated at room temperature for 25 min. After incubation, the beads were washed once with BW buffer and twice with 1×T4 ligase buffer. Finally, the beads were resuspended in 50 μl of DNase free water for subsequent library construction.

### DNA library construction

A commercial DNA library construction kit [VAHTS Universal DNA Library Prep Kit for Illumina V4 (ND610)] was used for library preparation. The main procedure consisted of four stages: DNA end repair, adapter ligation, library amplification, and library purification. Following adapter ligation, beads were washed three times with BW buffer to reduce adapter-related background. Starting from approximately 150 ng of adaptor-ligated DNA, the library was amplified using 9–10 cycles of PCR with MGI indexing primers. After 10 cycles, we typically obtained around 1300 ng of final library DNA, which was sufficient for high-throughput sequencing. Finally, the DNA library was purified using 0.9× DNA clean beads. To adapt to the MGI DNBSEQ-T7 sequencing platform, all adapters and amplification primers were replaced with their MGI-compatible counterparts.

### Data analysis

Micro-C data were processed using Microcket software (version 1.4) with default parameters [27]. Raw contact maps were used for all downstream analyses without balancing. The resulting pair files were then converted to “.hic” format using Juicer Tools (versions 1.9.9 or 1.19.02) [28]. Subsequently, the .hic files were converted to “.cool” files at multiple resolutions using HiCexplorer (version 3.7) [29].

## Results

### Titration of MNase concentration with 100 000 cells

**Figure 1:**
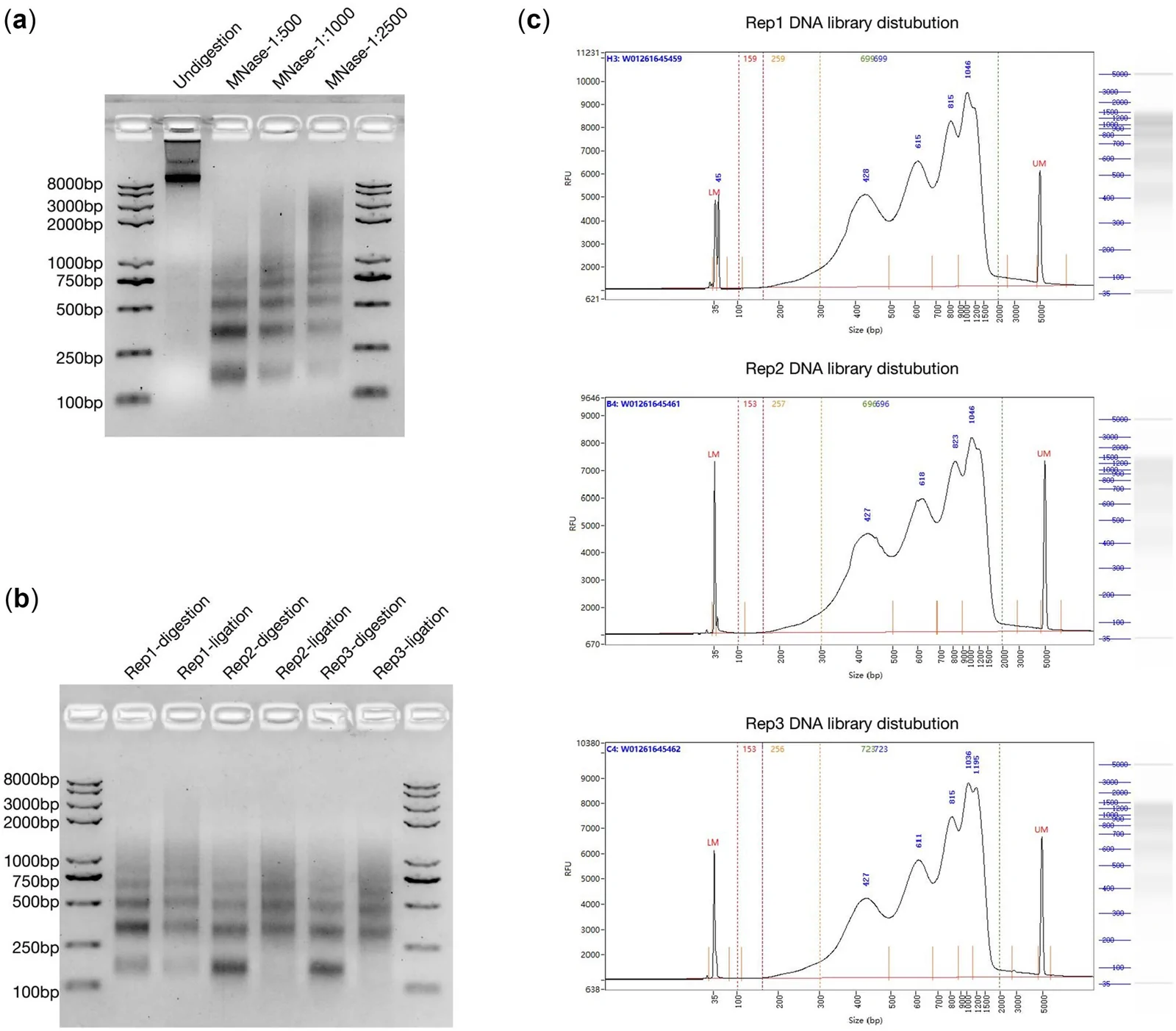
High-quality mononucleosomal DNA fragmentation from 0.1 million cells. (a) Agarose gel electrophoresis showing MNase titration. Lane 1: undigested DNA (no MNase); lanes 3–5: DNA digested with increasing concentrations of MNase. (b) Agarose gel electrophoresis depicting digestion and ligation efficiency for three replicate samples. (c) Bioanalyzer trace of the final Micro-C library. The library shows peaks from 400–1000 bp (RFU 5000–10 000, peak at 1000 bp), with 0.8× bead purification removing mono-nucleosomal fragments (∼150 bp).

To determine the optimal MNase concentration for digesting chromatin from 100 000 cells, we performed a titration of MNase on G1E-ER4 erythroblast cells [30]. Agarose gel electrophoresis revealed that, compared with 1:1000 and 1:2500 dilutions, a 1:500 dilution of MNase generated abundant mononucleosomal DNA fragments along with moderate amounts of oligonucleosomal fragments, which are suitable for subsequent ligation steps and 3D chromatin architecture analysis (Fig. 1a). After confirming the appropriate MNase concentration, we conducted three biological replicates on G1E-ER4 cells, further validating that the digestion pattern obtained with this dilution yields DNA fragments compatible with downstream library construction and data analysis (Fig. 1b). Additionally, to streamline the experimental workflow, we simplified the conventional multi-step T4 ligation to a one-step ligation, which exhibited high efficiency (Fig. 1b).

The final sequencing library was analyzed on a Bioanalyzer. As shown in Fig. 1c, the library displayed peaks from 400 bp to 1000 bp (RFU 5000–10 000, peak at 1000 bp). A 0.8× bead purification was used to remove mono-nucleosomal fragments and preserve library complexity; no peak at ∼150 bp was observed. Starting from ∼150 ng of adaptor-ligated DNA, 9–10 PCR cycles yielded ∼1300 ng of library DNA, sufficient for sequencing.

Overall, a 1:500 dilution of MNase is optimal for digesting 100 000 G1E-ER4 cells, and the resulting library meets the quality requirements for Micro-C analysis.

### Low-input Micro-C captures A/B compartments, TADs, and loops from 100 000 cells

To demonstrate the robustness and feasibility of Micro-C data obtained from 100 000 cells, we performed a detailed comparison between our dataset and a publicly available Micro-C dataset [23] (hereafter referred to as “5 million cells”) generated from the same G1E-ER4 cell line. To ensure fair comparison, we downsampled both datasets to 120 million raw read pairs. The key quality statistics are summarized in Table 1. Our low-input dataset achieved a cis ratio of 96.1% and a duplication rate of 3.0% (after 9–10 PCR cycles), indicating high library complexity and low background noise.

**Table 1:** Sequencing and mapping statistics of the low-input micro-C data.

	5 million cells		100 000 cells	
No. of category	Count	Fraction (%)	Count	Fraction (%)
**No. of preprocessing and alignment**				
** Total**	125 000 000	100	125 000 000	100
** Ktrim**	123 030 998	98.4	124 999 895	100
** Unique**	97 986 235	79.6	121 255 373	97
** Deduplication**	23 115 280	18.8	3 744 522	3
** Mappable**	75 746 390	77.3	104 585 246	86.3
**No. of interactions**				
** Uncalled**	2 289 845	3	8 175 761	7.8
** Incomplete-mapping**	681 916	0.9	1 715 187	1.6
** Too-many-segments**	0	0	623 227	0.6
** Unpairable**	1 583 832	2.1	5 748 076	5.5
** Self-circle**	24 097	0	89 271	0.1
** Reported**	73 456 545	97	96 409 485	92.2
** Cis (<1K)**	24 190 853	31.9	48 498 419	46.4
** Cis (1–10K)**	17 971 923	23.7	17 768 935	17
** Cis (≥10K)**	26 263 302	34.7	26 044 955	24.9
** Trans**	5 030 467	6.6	4 097 176	3.9

**Figure 2:**
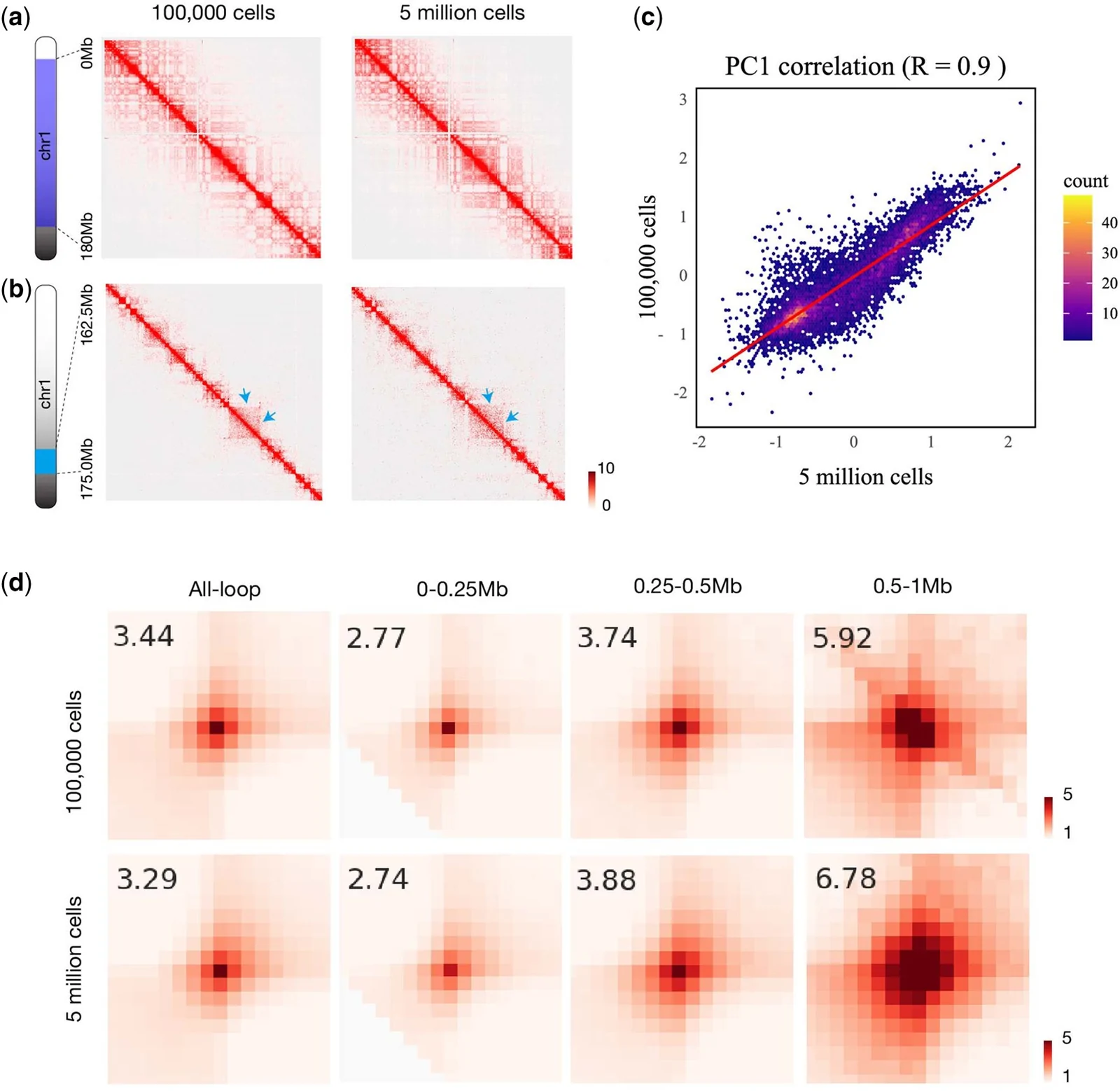
Low-input Micro-C captures A/B compartments, TADs, and loops from 100 000 cells. (a) Hi-C contact maps at 250 kb binning showing a comparison of compartmentalization between 100 000 cells and 5 million cells. (b) Hi-C contact maps at 25 kb binning showing a comparison of TAD strength between 100 000 cells and 5 million cells. (c) PC1 values (250-kb resolution) from 100 000 cells plotted against those from 5 million cells. Pearson correlation coefficient *R* = 0.9, indicating highly concordant a/B compartment profiles between the two datasets. (d) APA comparison of loop enrichment between 100 000-cell and 5 million-cell datasets (downsampled to 120 M raw reads). The loop strengths were comparable between the two conditions.

Examination of A/B compartments at 250 kb resolution showed that both datasets exhibited clearly discernible compartment patterns (Fig. 2a). To quantitatively assess their similarity, we compared PC1 values and obtained a Pearson correlation coefficient of 0.9 (Fig. 2c), indicating a high degree of concordance. At a finer scale, we inspected topologically associating domains (TADs) at 25 kb resolution. The TAD boundaries observed with 100 000 cells coincided well with those identified using 5 million cells, although the latter displayed slightly sharper domain definition (Fig. 2b).

**Figure 3:**
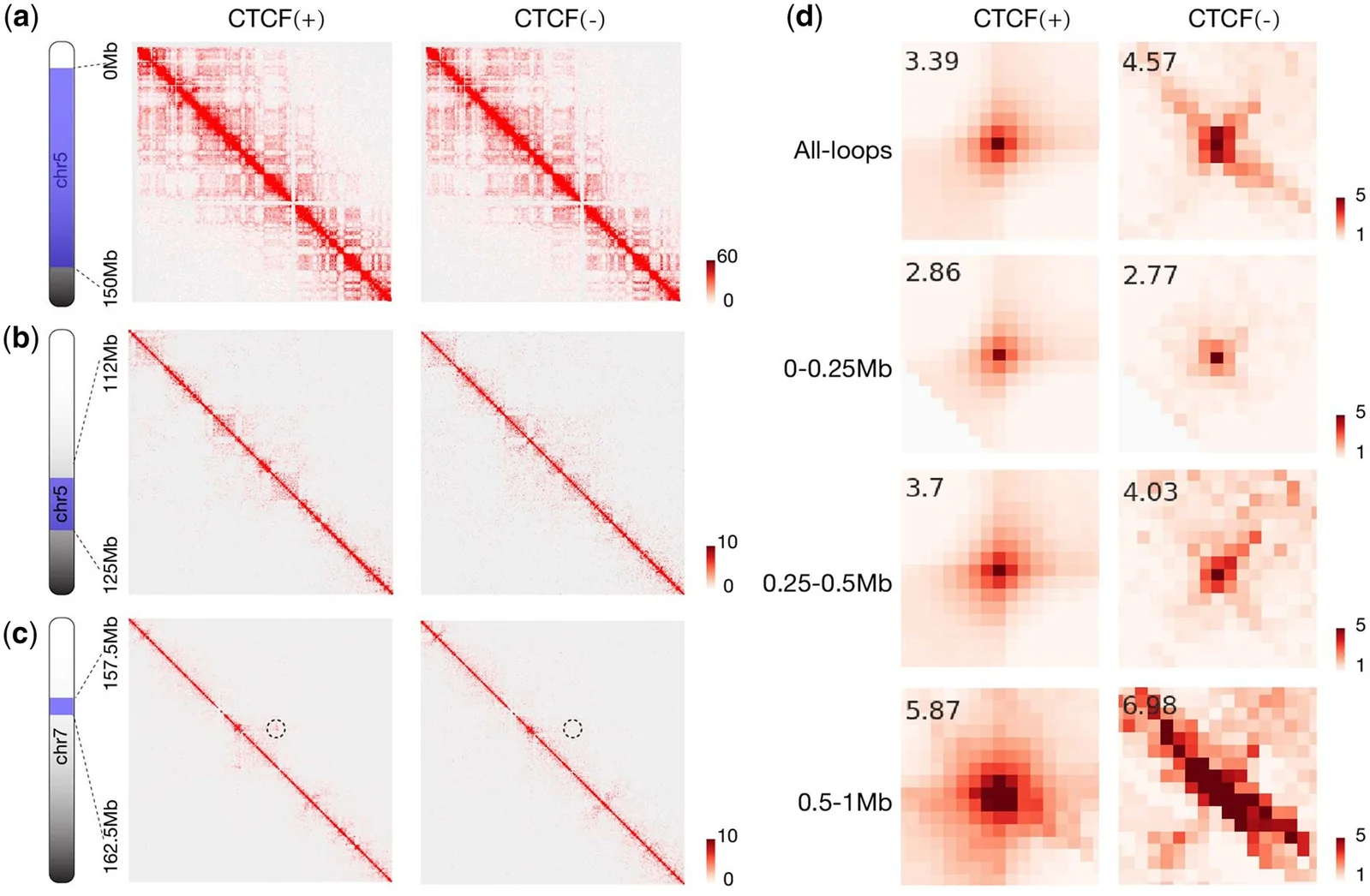
Low-input Micro-C sensitively detects loss of loops and TADs upon CTCF degradation. (a) Hi-C contact maps at 100 kb binning showing a comparison of compartmentalization between CTCF (+) and CTCF (−). (b) Hi-C contact maps at 25 kb binning showing a comparison of TAD strength between CTCF (+) and CTCF (−). (c) Hi-C contact maps at 25 kb binning showing a comparison of loop strength between CTCF (+) and CTCF (−). (d) APA reveals that chromatin loops are substantially enriched at CTCF (+), whereas chromatin loops are nearly undetectable at CTCF (−).

We further compared chromatin loops at 25 kb resolution. Notably, the same loop structures were clearly discernible in both datasets. Aggregate peak analysis (APA) was performed after downsampling both datasets to the same number of raw read pairs (120 M). As shown in Fig. 3d, the APA scores for the low-input sample were comparable to those of the control across different distance ranges, confirming that the simplified low-input protocol does not compromise loop detection.

In summary, Micro-C experiments performed with as few as 100 000 cells can capture key chromatin features, including compartmentalization, TADs, and various loop structures. Although the data coverage is lower than that obtained from 5 million cells, our results demonstrate the feasibility of applying Micro-C to low-input samples of 100 000 cells.

### Low-input Micro-C sensitively detects loss of loops and TADs upon CTCF degradation

To further validate the applicability of low-input Micro-C, we investigated the role of CTCF in 3D genome organization upon acute protein degradation. CTCF-AID-HaloTag cells (generated by our group in a separate study) were treated with 5-ph-IAA for 4 h to induce CTCF degradation. Micro-C libraries were then prepared from 100 000 cells. Comparative analysis revealed that upon CTCF degradation, loop signals at corresponding genomic loci were diminished (Fig. 3d) and TAD boundaries were lost (Fig. 3b and c), whereas A/B compartments remained unaffected (Fig. 3a). These observations are consistent with previous studies [26]. Collectively, our results demonstrate that Micro-C data obtained from as few as 100 000 cells can robustly capture the impact of CTCF depletion on 3D architecture of chromatin.

## Discussion

In this study, we developed an optimized low-input Micro-C protocol requiring only 0.1million cells, substantially reducing the cell requirement compared to standard methods [12, 20–25]. Our optimization focused on two critical steps: precise titration of MNase to achieve optimal nucleosome-resolution fragmentation (1:500 MNase: H_2_O ratio) and simplification of the ligation step to a one-step reaction, both of which were validated by agarose gel electrophoresis showing high-quality fragment distribution and efficient ligation.

A systematic comparison between low-input (100 000 cells) and standard-input (5 million cells) datasets, each sequenced to 30 G depth, revealed important insights. While the clarity of compartment and TAD boundaries was reduced in low-input data—likely due to decreased interaction matrix density—all key architectural features were faithfully preserved. This demonstrates that our protocol captures biologically meaningful information rather than noise. APA analysis after downsampling to equal valid pairs showed that the low-input protocol produced loop enrichment scores comparable to the control across all distance ranges (Fig. 2d), confirming that the simplified low-input workflow does not compromise the detection of chromatin loops. This observation warrants further investigation but underscores the robustness of loop detection in our optimized protocol.

The biological utility of our method was validated through acute CTCF degradation experiments using only 0.1million cells per condition. Consistent with previous Hi-C studies [26], CTCF depletion led to loss of loops and TAD boundaries, confirming the well-established role of CTCF in loop formation. Importantly, the ability to perform these experiments with low cell input enabled us to examine acute degradation dynamics without extensive cell expansion, which could otherwise alter chromatin states. This demonstrates that our protocol is not merely a technical achievement but a practical tool for addressing biological questions in sample-limited scenarios.

Despite these advances, certain limitations remain. The reduced clarity of higher-order structures suggests that analyses requiring extremely fine boundary resolution may still benefit from higher cell inputs. Additionally, detection of very weak or rare interactions may be compromised. Future improvements could incorporate unique molecular identifiers to distinguish biological signal from PCR duplicates better, or extend this approach to single-cell Micro-C. Nevertheless, our optimized protocol substantially expands the accessibility of nucleosome-resolution 3D genome mapping to rare cell populations, enabling a wide range of investigations into chromatin dynamics in development, disease, and beyond.

## Data Availability

The raw sequencing data for the low-input Micro-C samples (100 000 cells) generated in this study have been deposited in the Gene Expression Omnibus (GEO) under accession number GSE322813. The publicly available Micro-C dataset for G1E-ER4 cells generated from 5 million cells (used as a control) was obtained from GEO under accession number GSE254373. All other relevant data are available from the corresponding author upon reasonable request.
